# Development of Viral Vectors for Use in Cardiovascular Gene Therapy

**DOI:** 10.3390/v2020334

**Published:** 2010-01-27

**Authors:** Paul D. Williams, Parisa Ranjzad, Salik J. Kakar, Paul A. Kingston

**Affiliations:** Vascular Gene Therapy Unit, School of Biomedicine, Manchester Academic Health Science Centre, Room 3.26, Core Technology Facility, 46 Grafton Street, Manchester, M13 9NT, UK; E-Mails: paul.williams-3@manchester.ac.uk (P.D.W.); parisa.ranjzad@manchester.ac.uk (P.R.); salik.kakar@postgrad.manchester.ac.uk (S.J.K.)

**Keywords:** cardiovascular gene therapy, adenovirus, adeno-associated virus, lentivirus

## Abstract

Cardiovascular disease represents the most common cause of mortality in the developed world but, despite two decades of promising pre-clinical research and numerous clinical trials, cardiovascular gene transfer has so far failed to demonstrate convincing benefits in the clinical setting. In this review we discuss the various targets which may be suitable for cardiovascular gene therapy and the viral vectors which have to date shown the most potential for clinical use. We conclude with a summary of the current state of clinical cardiovascular gene therapy and the key trials which are ongoing.

## 1. Introduction

Although cardiovascular disease is the second most frequently targeted indication in clinical trials of gene therapy, with 137 such studies having received approval by the early part of 2009 (http://www.wiley.co.uk/genmed/clinical/), this is a distant second to studies of cancer-related pathologies, which account for almost 65% of gene therapy clinical trials with close to 1,000 trials initiated or approved at the time of writing. However cardiovascular disease is the most common cause of mortality in the developed world, primarily as a result of obstructive atherosclerosis of the coronary and peripheral arteries, and is associated with an enormous symptom burden, manifesting most commonly as angina pectoris and intermittent claudication when affecting the coronary and lower limb arteries, respectively. Plaque rupture of advanced atherosclerotic lesions leading to acute arterial occlusion is the usual aetiology of myocardial infarction and stroke, which are the commonest causes of mortality associated with atherosclerosis. Heart failure due to left ventricular dysfunction, itself frequently a consequence of myocardial infarction, is increasing rapidly in prevalence in an aging population, partly because of improved therapies for the acute phase of myocardial infarction that result in greater rates of survival from the actual event and more long-term morbidity as a consequence.

At least part of the reason for the relative paucity of clinical studies of gene therapy for cardiovascular diseases resides in the fact that reasonably successful “classical” treatments exist for many cardiovascular pathologies. Substantial symptomatic relief from obstructive atherosclerotic disease affecting the coronary or peripheral arterial trees, for example, can be afforded by balloon angioplasty (with or without stent implantation) or by bypass surgery, but these interventions are in most instances simply symptomatic rather than curative, with no modification of the underlying disease process. Furthermore, such treatments, while having the potential to relieve to a substantial degree the symptoms incumbent upon cardiovascular pathologies, do so at the cost of giving rise to what are in effect a new range of cardiovascular pathologies, including restenosis, stent thrombosis and saphenous vein bypass graft disease, which can result in a recurrence of symptoms in a relatively short time frame. Some pathologies, heart failure being a prime example, are not typically suitable for such interventional approaches to management and rely largely upon pharmacological treatments aimed at symptomatic alleviation, risk factor modification and suppression of disease progression. Although the prognosis for heart failure has improved as a result of such medical therapies, and the use of implantable cardiac devices in carefully selected patients, the condition is still associated with significant morbidity and mortality.

Set in this context, gene therapy has the potential to deliver novel therapies for diseases of the cardiovascular system (CVS) and numerous gene therapy approaches have been investigated to target the different manifestations of cardiovascular disease. These include therapeutic angiogenesis to relieve ischaemia due to severe coronary artery disease (CAD) or peripheral arterial disease (PAD) not amenable to surgery or percutaneous interventions; reducing neointimal hyperplasia (NIH) to prevent accelerated forms of atherosclerosis in stented arteries and in venous bypass grafts; improving cardiomyocyte function for the treatment of heart failure, and providing a long-term treatment for chronic multifactorial cardiovascular disorders such as hypertension and dyslipidaemias. In this review we will discuss the most commonly investigated clinical applications for cardiovascular gene therapy and the potential vector delivery mechanisms for each of these. We will then consider the viral vectors which currently show the most promise for use within the CVS and give an overview of the current field with regards to clinical studies.

## 2. Potential Targets for Cardiovascular Gene Therapy

As alluded to already, the CVS possesses numerous possible targets for gene therapy. These are outlined in Table 1. Clearly therapeutic approaches to cardiovascular disease are not of necessity targeted to cardiovascular tissues; however most research has focused on eliciting transgene expression in either the vascular wall or the myocardium. Delivery of gene transfer vectors to the vasculature or to the heart presents different technical challenges, and the precise nature of these challenges varies in accordance with the specific pathology that is targeted.

### 2.1. Vascular Gene Therapy

The vascular wall consists of three layers: the intima, a single layer of endothelial cells (ECs) that lie on the luminal surface of the vessel, overlying a thin layer of connective tissue; the media, consisting of vascular smooth muscle cells (SMCs) and connective tissue; and the adventitia which consists predominantly of loose connective tissue, but also contains fibroblasts. These layers are separated by the internal and external elastic laminae respectively. Gene transfer into the vascular wall was demonstrated first in 1989 [1]. Porcine primary ECs were transduced *ex vivo* using a murine amphotropic retrovirus and subsequently reintroduced into isolated segments of porcine ileo-femoral arteries using a double-balloon catheter. Since proof of principle was established the main clinical problems that have been investigated as potential targets for vascular gene therapy are prevention of restenosis post-coronary angioplasty (which now occurs principally in the guise of in-stent restenosis following coronary stent deployment), saphenous vein graft (SVG) degenerative disease following coronary artery bypass grafting (CABG), and induction of therapeutic angiogenesis within the peripheral and coronary arterial trees.

#### 2.1.1. Restenosis

**Figure 1.**
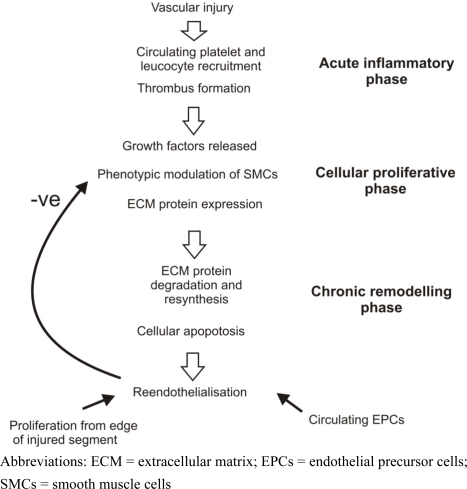
Flow chart illustrating the restenotic process.

Since the procedure was first performed in 1977, percutaneous coronary intervention (PCI, also known as angioplasty) has become the predominant method of revascularisation for patients with symptomatic coronary disease. In brief, PCI is performed under local anaesthetic with vascular access obtained via the femoral or radial artery. A catheter is inserted into the ostium of the target coronary artery and a fine guide wire is passed across the stenosis. Further catheters may then be advanced over the guide wire. The stenosis is typically dilated with a balloon followed by implantation of a stent: a metallic expandable coil which is mounted onto a second balloon to allow deployment. Since the mid-1990s, when stent deployment became a routine part of the PCI process, the number of cases has increased dramatically and, in the present day, several million such procedures are performed worldwide each year. However in-stent restenosis (ISR), progressive luminal narrowing as a result of neointimal hyperplasia within the stent, requires further treatment in approximately 14% of patients undergoing PCI with bare metal stents [2]. Neointimal hyperplasia occurs as part of the vascular healing response and is thought to arise primarily as a result of SMC migration (and perhaps proliferation) and extracellular matrix (ECM) deposition (see Figure 1). In humans the process is generally complete within six months of bare metal stent implantation at which time the stented segment of vessel has usually fully re-endothelialised. The widespread use of drug-eluting stents (DES), coated with potent anti-mitotic agents, has reduced the incidence of ISR but is associated with a new set of problems related to delayed vascular healing and inadequate re-endothelialisation of the metal stent struts. This includes late stent thrombosis: a sudden and life-threatening event. In addition, there is evidence to suggest that DES in current clinical use merely delay the onset of ISR rather than abolishing it completely [3]. There is, consequently, unequivocal scope for improved methods of percutaneous treatment of obstructive CAD.

Gene therapy strategies to attain this goal include reducing SMC proliferation and migration, inhibiting thrombosis, reducing ECM deposition and enhancing endothelialisation. Coronary stents provide a very convenient platform for delivering viral vectors [4–6]. Stents remain in the vessel wall permanently and allow sustained local exposure of the vessel wall to the gene transfer vector at the exact site of pathology whilst minimizing the risk of non-target-organ transduction. Stent deployment is performed with a high pressure balloon which causes localised vessel wall injury which results in two additional benefits with regards to gene transfer. Firstly, endothelial denudation removes a potent barrier to viral transfer. Secondly, quiescent medial SMC transform to a proliferative phenotype as a response to injury which can increase gene transfer efficiency due to breakdown of the nuclear envelope. Several groups have reported successful virus-mediated gene transfer from stents in pre-clinical studies with potentially therapeutic effects *in vivo* [7–9].

#### 2.1.2. Saphenous Vein Graft Degeneration

Coronary artery bypass grafting (CABG) is one of the most commonly performed surgical procedures worldwide, and in spite of the use of arterial grafts where possible, autologous saphenous vein is still the most widely used conduit for CABG [10]. The poor long-term patency of vein grafts is a major problem: historically around half of all vein grafts are occluded ten years post-CABG and half of the remainder are severely diseased [11–13]. Using contemporary surgical techniques vein graft failure at 12–18 months is still approximately 25% and around 40% of patients undergoing CABG will have at least one of their grafts fail at this time point [14]. As with ISR, vein graft failure is usually a consequence of NIH [15–17] particularly in the first 12 months post-implantation. Neointima also promotes development of superimposed atherosclerosis which can lead to graft failure over the longer term. Part of the allure of the prevention of vein graft failure as a target for gene therapies lies in the dearth of extant preventative pharmacotherapies (although vigorous cholesterol lowering does delay vein graft atherosclerosis [18]), but the primary advantage of targeting venous bypass conduits lies in the availability of vein segments for *ex vivo* gene transfer during their harvesting and preparation for implantation, which offers immense ease of local delivery without the need for complex technologies as is required for ISR prevention. This is offset to an extent by the limited time available for gene transfer; a vein segment might be used within minutes of harvesting, which contrasts with the permanent platform for vector transfer that is offered by a coronary stent.

#### 2.1.3. Angiogenesis

Although advances in surgical and catheter-based techniques now allow revascularisation for the majority of patients with symptomatic CAD and PAD, there exists a proportion of patients in whom these techniques are not applicable either because of excessive procedural risk or because of technical difficulties related to the arterial anatomy. PCI and CABG allow treatment of coronary vessels with a diameter of approximately 2 mm or greater (macrovascular disease), but a significant minority of patients have microscopic coronary disease which is unsuitable for revascularisation by conventional means. Such patients have been studied in many early clinical trials wherein gene therapeutic approaches have utilised pro-angiogenic transgenes in attempts to afford symptomatic alleviation. The induction of therapeutic angiogenesis aims to increase blood flow to ischaemic tissue by the generation of new blood vessels. In the peripheral vasculature, the aim of such therapy is typically the relief of limb pain occurring at rest and the prevention of limb loss in patients with critical ischaemia. In the coronary vasculature, as well as improving exertional angina, coronary angiogenesis has the potential to improve left ventricular function in patients with heart failure: chronically ischaemic regions of myocardium may have poor contractility as a result of hibernation of viable myocardial cells (cardiomyocytes) and these cells may regain normal function with improvement of regional blood flow. As a consequence of the clinical need that these conditions represent – being akin to advanced malignancies in terms of the symptomatic severity and the mortality with which they are associated - they are at the time of writing the most extensively studied application for cardiovascular gene transfer. As well as arterial catheter infusion, direct injection of virus either into skeletal muscle for PAD or into the myocardium at the time of CABG or via a mini-thoracotomy for CAD has been investigated.

### 2.2. Myocardial Gene Therapy

Gene delivery to cardiomyocytes, principally within the left ventricle, offers the potential to treat several conditions. As discussed above, pro-angiogenic genes may improve blood supply to the myocardium, and localized delivery of genes involved in the generation and propagation of cardiac electrical activity offers the potential for “biopacemaking” as an alternative to permanent implantable electronic pacemakers [19]. Myocardial gene therapy may also be useful for the cardiovascular manifestations of genetic conditions such as Fabry disease [20] and ion channel disorders (for instance single gene long QT syndrome). At the present, however, heart failure is the most actively investigated potential target for myocardial gene therapy.

#### Heart Failure

Heart failure is a clinical syndrome characterized by shortness of breath on exertion, fluid retention and fatigue. It typically occurs as a consequence of left ventricular dysfunction and its prevalence is reaching epidemic levels as the population ages: currently it is estimated that one in five 40 year olds will develop heart failure within their lifetime [21]. Management of the condition is complex and expensive, with an estimated cost of $37.2 billion for 2009 in the USA alone [21] and, despite improvements in pharmacological therapy and the increasing use of implantable cardiac devices that can improve left ventricular contractile function and reduce the risk of arrhythmic sudden death, the prognosis for patients with severe heart failure remains poor. In the CARE-HF study the mortality rate, at a median follow-up of 29 months, was 20% in patients with severe heart failure despite optimal medical therapy and biventricular pacemaker implantation [22]. Heart failure is a heterogeneous condition with multiple aetiologies, but two causes account for the majority of cases of heart failure in the developed world, of which the most common is ischaemic heart disease which leads to left ventricular dysfunction as a result of myocardial infarction and chronic ischaemia, and which usually leads to regional myocardial dysfunction. The other common cause is a primary disease of the myocardium known as dilated cardiomyopathy which has several potential aetiologies; it may occur subsequent to an infective precipitant, or in association with autoimmunity or pregnancy, but is most often idiopathic and manifests as global left ventricular dysfunction [23]. Irrespective of aetiology, the goal of gene therapy is to improve cardiomyocyte function in areas of myocardium which have reduced or absent contractility. An increased understanding of the pathology of heart failure at the cellular and molecular level has led to the identification of several potential molecular targets for gene therapy. These targets are primarily involved in either cardiomyocyte calcium handling or β-adrenoceptor signalling and have been reviewed recently [24]. Clinical trials using an adeno-associated virus (AAV) vector have begun and will be discussed in detail later on in this article.

Although heart failure is often considered a permanent progressive condition, significant cardiac dysfunction has been shown to completely resolve in some cases with the temporary use of left ventricular assist devices (LVADs) [25]. Short-term duration of transgene expression may therefore be sufficient to result in significant improvements in cardiac function. Delivery mechanisms for cardiac gene therapy have recently been reviewed [26]: primary methods include intramyocardial injection, intrapericardial injection and intracoronary infusion. Although the intravenous route represents the most convenient method of administration, and has shown potential in rodents [27], huge virus doses would be required in humans, which would be difficult with current production techniques and would pose safety issues. The ongoing human trials of AAV are using catheter-mediated coronary infusion for vector delivery.

### 2.3. Other Targets

Other putative therapeutic applications of cardiovascular gene therapy include risk factor modifications such as cholesterol lowering or antihypertensive gene therapy. Both high blood pressure and hypercholesterolaemia typically require lifelong oral therapy at the present and while such therapies are often very effective, the potential for gene therapy to act as a one-shot treatment for these chronic pathologies makes them attractive targets for investigation. However we shall confine ourselves for the remainder of this review to a consideration of those viral gene transfer vectors that have been applied to or have potential application to clinical cardiovascular gene transfer, and to a discussion of the current state of clinical virally-mediated gene therapy in the CVS.

## 3. Virus Vectors for Cardiovascular Gene Transfer

The ideal vector for clinical application would be target cell-specific with no expression outwith the target cell type; offer the capacity to transfer large DNA sequences; result in therapeutic levels of transgene expression that are not attenuated by the host immune response; express transgene for a duration appropriate to the clinical problem; pose no risk of toxicity either acutely (as a result of immunogenicity or unregulated transgene expression) or in the long-term (such as oncogenesis); and be cost-effective and easy to produce in therapeutically applicable quantity. Clearly no currently available vector fulfils this wish-list of characteristics, and it is probably unrealistic to suggest that such an ideal vector will ever exist. Several viruses have been considered for use in cardiovascular gene therapy and all represent some compromise of the above features. Adenovirus is the most commonly used virus in clinical trials of cardiovascular gene therapy to date (http://www.wiley.co.uk/genmed/clinical/), although recent advances in the development of recombinant AAVs have led to the initiation of clinical trials using this vector for the treatment of heart failure. Lentiviruses have yet to be used in cardiovascular clinical trials, as a result of concerns over long-term safety, but the recent development of non-integrating lentiviruses may make this vector an appealing option in the future. Other retroviruses, sendaivirus, Semliki forest virus, herpes simplex virus and baculovirus [28–33] have all undergone pre-clinical investigation for cardiovascular gene therapy, but have important limitations and have never been subject to clinical trials. In the next sections we will focus, therefore, on the three viral vectors that we regard (and it is probably true to say are widely regarded by others too) as showing the most promise for clinical use in cardiovascular disease: adenovirus, AAV and lentivirus.

### 3.1. Adenovirus

Adenoviruses (Ad) were first described in 1953 [34]. Adenovirus is a natural human pathogen and over 50 serotypes of human adenovirus are known to exist [35,36]: wild-type infection most commonly causes respiratory tract infections, but can also result in pharyngitis, gastroenteritis, conjunctivitis, haemorrhagic cystitis and, most importantly from our perspective, myocarditis. Indeed, adenovirus infection (including infection by serotype 5 adenovirus, which is the basis of the most commonly used recombinant adenovirus vectors) is one of the commonest viral causes of acute myocarditis in children and young adults [37]. As anyone familiar with virus-mediated gene transfer will be aware, adenoviruses have several features which make them attractive for gene therapy: they have a broad natural tropism (reflected in the variety of illnesses that they can cause in wild-type guise); their high nuclear transfer efficiency ensures a rapid onset of transgene expression; they do not integrate into the host genome and do not, therefore, carry an appreciable risk of oncogenesis; they can infect both dividing and quiescent cells, and they can easily be produced in large quantities. The principal disadvantage of adenoviruses is their potent pro-inflammatory nature. This is largely a consequence of the hit-and-run fashion of wild-type adenovirus infection: adenoviruses have no mechanisms of cellular persistence and rely upon infecting and rapidly producing large quantities of new virus from host cells before they are killed by host inflammatory responses. The E3 region of the adenovirus genome encodes proteins that assist in evading host immunity, but these do so only to such an extent that will allow infected cells to survive long enough post-infection for the adenovirus lytic cycle to complete. The pro-inflammatory nature of adenoviruses also results in a limited duration of transgene expression as a consequence of clearing of infected cells by host inflammatory and immune mechanisms (although this may, in fact, be advantageous in certain applications where the pathological process is transient, such as neointima formation following coronary stent deployment). Their widespread prevalence as pathological agents in human communities also means that the majority of human adults have pre-existing adenovirus-neutralising antibodies [38]. Adenoviruses are also liable, consequent upon their broad tropism, to transduce non-target organs.

The use of adenoviruses for gene therapy has been reviewed numerously over the last 15 years and it would not serve us well to spend much of the present review discussing the basics of adenovirus biology from this perspective. However, briefly, the adenovirus virion consists of a non-enveloped icosahedral capsid particle containing a 30–40 kb linear dsDNA genome. Located at each of the twelve vertices of the icosahedron is a trimeric fibre shaft terminating in a globular knob domain. The length and flexibility of the fibre shaft varies significantly between adenovirus subtypes and can influence both binding and virus uptake [39]. The primary cell surface receptor for Ad5 is the Coxsackie-Adenovirus receptor (CAR) which binds to the knob domain and greatly enhances infection of cells upon which it is expressed [40]; heparan sulphate proteoglycans (HSPGs) and integrins act as co-receptors for certain cell-types. Not all cardiovascular cells express CAR, and while it is present on the surface of cardiomyocytes [41] it does not occur (or occurs only at low levels) on vascular SMCs and ECs [42], which are the commonest cell types in the vascular wall. The CAR is not essential for Ad5 infection however, and it is now appreciated that Ad cell-binding is more complex than previously thought. Alternative mechanisms of Ad5 transduction have recently been demonstrated *in vivo*: blood factors including coagulation protein IX and complement protein C4BP have been shown to bind the adenoviral fibre and promote localisation of adenovirus to the liver via cellular HSPGs and the LDL receptor proteins [43,44]. Other adenovirus serotypes have different primary receptors which are not as well characterised (see [45]). Following cell binding, the adenovirus virion enters the cell *via* clathrin-mediated endocytosis and undergoes endosomal processing prior to cytoplasmic release and delivery of the virus genome to the nucleus. Binding to the nuclear pore complex allows rapid transfer of the genome to the host nucleus [46]. Adenovirus is non-integrative and the genome remains in the nucleus in linear episomal form following successful infection.

Despite its broad natural tropism, when given systemically virtually all Ad5-mediated transduction occurs in the liver, predominantly within resident Kupffer cells [47]. Combined with the low-level of CAR expression on ECs, this makes Ad5-derived vectors poor candidates for systemic administration to the vasculature. However, Ad5 can transduce ECs *in vivo* if administered locally [48,49] and, although under normal circumstances the endothelium represents a barrier that adenoviruses cannot easily cross (except in the liver) [50], Ad5 can transduce medial SMC effectively if there is endothelial denudation [49], which occurs in association with advanced atherosclerosis and at sites of PCI (as a result of the physical process of intervention). Adenovirus is also capable of very effective myocardial transduction after local delivery; almost 80% of cardiomyocytes were transduced following combined simultaneous transfusion of first generation vector into the left anterior descending coronary artery and great cardiac vein in juvenile pigs [51]. As a consequence, Ad5 has found use in cardiovascular gene transfer in studies of localized delivery of gene therapies to the vessel wall, to the myocardium and into skeletal muscle in ischaemic limbs.

Recombinant vectors derived from serotype 5 adenovirus (Ad5 – a subgoup C adenovirus) are by far the best characterised and have been used in the majority of clinical trials. First-generation recombinant Ad5 have typically had the E1 and E3 regions (which contain genes that are essential for viral assembly and for evasion of host immunity respectively) removed. Several second-generation recombinant Ad5 have been described that include additional deletions of the adenovirus genome from the E4 locus [52], or the E2A region [53]. Other second-generation modifications include functional mutations in the E2A region or the inclusion of an immunomodulatory transgene from the serotype 2 adenovirus [53]. Some evidence exists to suggest that modest benefits in transgene expression might be obtained by use of such second-generation vectors in preference to first-generation adenoviruses within the vasculature [52], although greater transgene expression was manifest only at 10 days after infection of rabbit carotids. No difference in transgene expression was observed at 3 or 28 days post-infection in this study, and other studies of second-generation vectors have provided no evidence at all of benefit in magnitude or duration of transgene expression after arterial gene transfer [53]. As a consequence, first-generation recombinant adenoviruses, despite (or perhaps because of) their relative simplicity, have remained the mainstay of clinical studies of gene therapy within the vasculature.

Further modification has given rise to third-generation recombinant adenoviruses, from which all wild-type adenoviral coding sequences have been deleted. These viruses have an increased cloning capacity of approximately 35 kb (compared to around 8 kb in first- and second-generation viruses) and produce no viral proteins in infected cells [54], as a result of which they give rise to markedly reduced host adaptive immune responses and longer durations of transgene expression [55]. These helper-dependent (or “gutless” if you prefer the more colourful nomenclature) vectors have been applied to gene transfer within the vasculature of animals with impressive medium-term results [56,57]. Transgene expression persisted for at least eight weeks in rabbit carotids infected with a helper-dependent adenovirus expressing rabbit urokinase-type plasminogen activator, with stable expression from day 14 to day 56, which contrasted with complete loss of transgene expression by day 14 from arteries infected with first-generation viruses. Helper-dependent adenoviruses also elicited a significantly reduced inflammatory response within rat myocardium compared with first-generation viruses, which was associated with evidence of prolonged transgene expression [58]. On these bases, it seems that gutless adenoviruses are superior to their first- and second-generation forebears as vectors for cardiovascular gene transfer. However, despite these reports, very few pre-clinical studies of cardiovascular gene transfer have used helper-dependent adenoviruses as their mode of gene transfer. They have been used in a murine model of hypertension in which tail vein injection of vector achieved long term (> 120 day) regulatable hepatic expression of atrial natriuretic peptide with concomitant reduction of heart weights and systolic BP in infected animals [59]. They have also been applied to a rabbit model of hindlimb ischaemia, in which intramuscular injection of a gutless adenovirus expressing sphingosine kinase resulted in improved limb perfusion 20 days post-delivery [60]. No more long-term observations were reported in this study however and, as no comparison was made with first- or second-generation adenoviruses, we will never know if this effect was greater than what might have been achieved by a more simple vector, or sustained for the months (or even years) that would be required to elicit an effect of genuine clinical value.

It is almost certain that part of the reason for the surprisingly limited uptake of gutless adenoviruses in pre-clinical studies of cardiovascular gene transfer lies in the relative difficulty of making the helper-dependent vectors. Most such pre-clinical studies are of a relatively short-term nature and it seems that the advantages in duration of transgene expression and reduced host inflammatory responses that are offered by gutless adenoviruses do not outweigh the extra effort of manufacture, particularly as Wen *et al* reported that peak transgene expression following helper-dependent virus-mediated gene transfer was only around 10% of that observed after a first-generation virus was used to deliver the same transgene [57]. In addition, gutless adenoviruses still induce an innate immune response to the viral caspid [61] (and possibly to CpG motifs within the viral genome itself [62–64]) and most adult humans still possess pre-existing antibodies to the serotype 5 virus particles: the absence of viral protein expression does not confer any greater capacity to evade pre-existing humoral immunity [57]. Until the advantages that third-generation adenoviruses undoubtedly possess in immunologically naïve experimental animals are shown to translate into clinical benefits by comparison to first-generation adenoviruses, it is likely that researchers will persevere with the old technology.

#### Reducing Immunogenicity and Improving Adenoviral Targeting

The immunogenicity of adenoviruses has proven to be a major stumbling block to their clinical use, both by limiting the magnitude and duration of transgene expression and by inducing dose-dependent toxicity. Their broad tropism is also an issue, particularly for systemically administered gene therapy. Both of these problems can potentially be circumvented by modifying Ad targeting *via* genetic alteration of viral proteins or by coating the virus with bi-specific molecules targeted at a component of the virus capsid and a specific host cell-surface protein. Adenoviral targeting for vascular gene therapy has been the subject of specific reviews in recent years [39,45].

Pseudotyping involves replacing the fibre components of a specific serotype of adenovirus with those from another serotype. Such a manoeuvre can reduce the immunogenicity of the chimeric product and redirect viral transduction to cell types for which the original adenovirus is not normally tropic. In practice, the initial vector that has been subjected to pseudotyping is always Ad5. Earliest reports of pseudotyping of Ad5 included the substitution of the Ad5 fibre head with that from the serotype 3 adenovirus (subgroup B), resulting in a chimera with alterations in tropism that included reduced transduction of human coronary ECs [65]. The same chimeric virus (Av9LacZ) was found subsequently to induce 10- to 15-fold greater transduction of human vascular SMCs from a variety of arterial beds than the parent Ad5 vector [66]. However transduction of pig and rat SMCs by Av9lacZ was reduced by comparison to the non-chimeric Ad5 progenitor. An even greater effect was observed when the Ad5 fibre was substituted with that from Ad16 (subgroup B). The resulting virus (Ad5.Fib16) gave rise to a 64-fold increase in transgene expression in umbilical vein SMCs along with an 8-fold increase in umbilical vein ECs although, once again, transduction of pig and rat SMCs was very substantially diminished [67]. Enhanced transduction was also observed in isolated segments of human coronary artery.

Ad5 has been pseudotyped with fibres from the subgroup D serotypes 19p and 37, resulting in chimeric vectors with very low tropism for hepatocytes compared with unmodified Ad5 [68]. Both Ad5/19p and Ad5/37 showed enhanced tropism for saphenous vein SMCs by comparison to Ad5, although Ad5/16 (similar to the virus Ad5.Fib16 investigated by Havenga *et al* [67]) showed very much greater enhancement of transgene expression in SMC. Reduced hepatotropism was much less marked in the case of Ad5/16 however, than was reported with Ad5/19p and Ad5/37.

Replacement of adenovirus components with peptides from different classes of virus to form chimeric viruses has been employed as an alternative strategy to pseudotyping [69], but the resultant viruses often have major structural defects [70].

An alternative approach to pseudotyping is modification of capsid components by selective mutation or by insertion of peptides to alter vector tropism. Mutation of the knob CAR receptor has been shown to reduce transduction of cells expressing CAR *in vitro* [71], however this was not sufficient to reduce hepatic transduction *in vivo* as a result of alternate transduction pathways [72]. The combination of mutations of the CAR receptor and the putative HSPG-binding site dramatically reduces hepatic transduction *in vivo* [72] but also appears to abolish infectivity in other cell types despite the insertion of a targeting peptide [73]. The insertion of targeting peptides into the H1 loop of the fibre knob has been employed to successfully retarget Ad5. The earliest attempts to retarget in such fashion involved the insertion of a cyclic RGD motif, which interacts with α_v_ integrins, resulting in a significantly increased transduction of cultured ECs and organ-cultured jugular veins from mouse, rat and rabbit, but no enhancement of SMC transduction [74]. A similarly modified virus was subsequently shown to significantly increase transgene expression in ECs and SMCs from human saphenous vein and to increase transduction of intact human saphenous vein segments [75]. Specific saphenous vein SMC-targeting peptides, identified by phage display, have also been inserted into the H1 loop. A short linear heptapeptide successfully enhanced adenovirus-mediated transduction of saphenous vein and coronary artery SMC, while detargeting the vector from ECs [76]. In the recent past, this approach has been taken a step further by the introduction of targeting peptides into the Ad19p fibre of a pesudotyped Ad5/19p virus resulting in a liver detargeted vector with octopeptide-mediated targeting to the heart [77].

Non-genetic means of transductional targeting involve coating the virus with a bi-specific adaptor which reduces the natural viral tropism and can be coupled to an antibody to selectively target a desired cell type. Examples of these bi-specific molecules include polymers such as polyethylene glycol (PEG) and antibodies. Coating of Ad with PEG coupled to anti-E-selectin-antibody has been shown to both prevent normal binding to the CAR receptor and to target ECs [78]. Although this approach appears to work *in vitro* a murine study showed that, although Ad PEGylation reduces the innate immune response, it does not affect the distribution or level of transduction suggesting alternative pathways of Ad transduction are important [64]. Bi-specific antibodies can also be used to redirect Ad tropism. Coating Ad with a bi-specific antibody targeting angiotensin-converting enzyme (ACE) specifically targets pulmonary vascular endothelium [79,80] and a similar approach has been shown in spontaneously hypertensive rats to reduce systemic blood pressure using a systemically administered adenovirus encoding for endothelial nitric oxide synthase [81]. Ad5 has also been targeted to SMCs and ECs using a bi-specific complex comprising the extracellular domain of CAR linked by an avidin-biotin bond to a cyclic RGD peptide, with concomitant enhancement of EC and SMC transduction by the targeted vector [42].

Despite significant advances in the understanding of adenovirus transduction and immunogenicity however, and the elegant means by which retargeting strategies have altered very significantly the tropism of the derivative adenoviruses, these targeted adenovirus vectors still remain laboratory tools and, thus far, none have made the transition into the clinical research setting. Furthermore, retargeting aside, these viruses are still basically serotype 5 adenoviruses and come with the problems of innate immunogenicity and pre-existing exposure to their wild-type progenitor in most human communities. As such, it is likely that the best that might be hoped for from these vectors in the state in which they exist now is transient expression of transgene (and consequently transient therapeutic effects) in a carefully targeted population of cells. It is likely that, before widespread clinical usefulness is attained, it will be necessary to further refine adenovirus technology to produce helper-dependent targeted vectors derived from serotypes other than Ad5. And even then, each vector is likely to be useful only once in each patient in whom it might be used. That may, of course, still be enough to be very useful indeed!

### 3.2. Adeno-Associated Virus (AAV)

AAV is a small member of the parvovirus family with a 4.7 kb single-stranded DNA genome. Wild-type AAV has three unique, potentially beneficial characteristics which distinguish it from other gene therapy vectors: firstly it cannot replicate without the assistance of a helper virus, such as adenovirus or herpes simplex virus; secondly the AAV genome is capable of long-term persistence within the nucleus, either by site-specific integration into the AAVS1 locus on the long arm of chromosome 19 or in episomal form; thirdly, despite the fact that a large proportion of the world’s population is seropositive for a variety of AAV serotypes, AAV has never been shown to cause human disease. Since the first infectious clone of AAV serotype 2 was established in 1982 [82], a total of 12 serotypes [83,84] and over 100 variants have been identified from human and non-human primate tissues [85]. Recombinant AAV (rAAV) vectors have had almost the entire viral genome removed, leaving only two regions of inverted terminal repeats (ITRs) in between which the transgenic DNA is inserted. The AAV Rep and Cap genes which are required for viral replication and packaging are supplied by a helper plasmid during the production process [86].

As a consequence of loss of the Rep gene, rAAV lose the capacity for site-specific integration into chromosome 19 and acquire the potential for random integration with the risk of oncogenesis, although the available evidence suggests that integration of AAV genomes is inefficient even in wild-type form and nuclear persistence is usually a consequence of episomal maintenance [87]. The actual risk of oncogenesis arising from random integration of AAV genomes is likely to be small therefore, although this is obviously a matter for future studies to clarify. Advances in the development of rAAV vectors have been reviewed in recent years [83].

Recombinant AAV offers some very advantageous features as a gene therapy vector. As wild-type AAV is not pathogenic it represents the safest of the viral vectors being considered and is significantly less immunogenic than Ad. Recombinant AAV elicit long-term gene expression as the genome persists in the nucleus, largely as circularised dsDNA episomes [88]. A single intramuscular injection of rAAV containing the factor IX gene to treat haemophilia B has been shown to result in continuing gene expression at 3.7 years in humans [89]. AAV are not without their drawbacks however. The onset of transgene expression is substantially delayed compared with other vectors, as a result of slow nuclear transport and the need for the single-stranded genome to be converted to dsDNA prior to expression [90]. As a consequence of this, early studies of AAV-mediated arterial gene transfer found no transgene expression within the first week following vessel infection, although transduction was manifest in the second week post-exposure [91]. AAV have been generated that contain a self-complementary double-stranded DNA genome. These elicit a significantly more rapid onset of transgene expression and enhanced cellular transduction by comparison to the parent ssDNA vector; however this improvement comes at the cost of a halving of the packaging capacity of the resulting vectors [92,93].

The small packaging capacity of AAV containing an ssDNA genome (approximately 4.6kb) imposes modestly severe limits upon the size of transgene expression cassette that can be inserted. Having said that, 4.6 kb still offers substantial scope for therapeutic gene transfer, and sterling work has been done in minimizing the size of therapeutic gene sequences in order to allow packaging into AAV. This is exemplified by AAV-mediated transfer of dystrophin: the full-length dystrophin cDNA at ≈14kb is far too large for packaging into AAV, yet a functional micro-dystrophin cDNA of 3.8kb has been packaged into a rAAV and used to elicit potentially therapeutic effects in mice [94]. Nonetheless, there are some genes that will probably never be suitable for AAV-mediated gene transfer, large ion-channels with multiple transmembrane regions for example, and physiological regulation of transgene expression from AAV by inclusion of genomic promoter sequences is likely to prove challenging. As with adenovirus, immune clearance of transduced cells can be a major problem too, particularly given the high prevalence of neutralising antibodies in the general population [95].

AAV2 was the first adeno-associated virus to be developed as a gene therapy vector and represents the most extensively investigated of the AAV serotypes. Infection is thought to be primarily mediated by membrane-associated HSPG [96], although other pathways for cellular uptake exist in non-hepatic tissue including the heart [97]. Removal of the HSPG primary receptor reduces liver transduction whilst cardiac transduction is preserved [98]. AAV2 is tropic for arterial SMCs and elicited transgene expression in 10–20% of medial SMCs 21 days after infection of rabbit carotid arteries *in vivo*. Endothelial transduction was poor however [49]. Comparison in cultured cells confirmed that AAV2 elicited modestly greater transduction of human saphenous vein SMCs than AAV3-8 and none of these alternative serotypes elicited substantial transduction of ECs either [99,100]. AAV2 targeting to increase SMC transduction has been achieved using the heptapeptide that was effective in targeting recombinant adenoviruses [76]: an increase of up to 70-fold in transgene expression was seen in human coronary artery SMCs exposed to targeted AAV by comparison with non-targeted vectors, although an 18-hour period of exposure was required for this magnitude of effect. A significant enhancement of transduction was observed in coronary artery SMCs after only one hour of exposure, although no enhancement of transduction of human saphenous vein SMCs was observed after this shorter period of exposure. Use of AAV for vascular gene transfer has been very limited however (the authors are aware of only one study that has ever attempted to elicit a ‘therapeutic’ effect by localized AAV-mediated vascular gene transfer [101]), and most interest in AAV within the cardiovascular system has been directed towards its use for myocardial gene transfer. In that respect AAV2 is not the most efficacious serotype for potential therapeutic application. AAV2 vectors pseudotyped with capsid proteins from other AAV serotypes have been studied to establish whether myocardial delivery can be improved by such means. AAV2 pseudotyped with AAV1, AAV6 and AAV8 capsid proteins all elicited greater myocardial transduction in rats than AAV2 after direct intramyocardial injection, at all time points up to 24 week post-infection [102]. AAV1 and 6 gave rise to transgene expression that maximized at four weeks and remained stable until the final 24-week time point. However, greatest expression at all time points was achieved by AAV8, which manifested an increase in transgene expression at each consecutive time point. In a different study, recombinant AAV2 pseudotyped with AAV1 (AAV2/1) increased transgene expression in human and adult murine cardiomyocytes by approx 2- to 3-fold when compared with AAV2 [103], but AAV2/8 and AAV2/9 were subsequently shown to elicit ≈20-fold and ≈200-fold greater myocardial transgene expression than AAV2/1 following intravenous injection into 1-day old mouse pups [27]. The cardiotropism of AAV9 was confirmed following intrapericardial injection into neonatal mice and adult rats, in which AAV9 produced global myocardial transduction that was stable for up to one year and significantly greater than AAV1, 6, 7 or 8 [104].

At the present, rAAV are the vector of choice for myocardial gene transfer and the capacity of serotypes 1, 6, 8 and 9 for effective transduction of cardiomyocytes offers the prospect of genuinely effective therapeutic myocardial gene transfer in the clinical setting. Unanswered questions remain about the prospect of integrational oncogenesis, and it is likely that the usefulness of rAAV as therapeutic agents will ultimately be confined by their limited capacity to deliver transgenic material. Nevertheless, rAAV offer the best prospect of breakthrough successes in the field of clinical virus-mediated cardiovascular gene therapy.

### 3.3. Lentivirus

Lentiviruses are part of the retrovirus family and consist of a ssRNA genome enveloped in a lipid bilayer; most currently investigated lentiviruses are derived from HIV-1. The primary receptor for lentivirus is the T-cell CD4 receptor and, as opposed to Ad and AAV, cellular entry occurs via membrane fusion. The viral capsid is subsequently released into the cytoplasm where uncoating and reverse transcription of the viral ssRNA to dsDNA occurs followed by nuclear transport via the microtubuli [105]. A major advantage of lentiviruses is that, unlike Ad and AAV, they are not inherently immunogenic. Unlike other retroviruses, which cannot readily cross the nuclear membrane, lentiviruses are able to transduce non-dividing cells, which is an attractive characteristic for cardiovascular gene therapy as vascular cells and cardiomyocytes are quiescent in their resting state. Lentivirus possesses an 8 kb packaging capacity.

Two major developments were required to make lentivirus a possible gene therapy vector. Firstly, self-inactivating lentivirus vectors were generated in which the U3 promoter region of the long terminal repeat had been inactivated [106], reducing the chance that homologous recombination and generation of wild-type HIV-1 can occur. Secondly, given that wild type lentivirus only infects CD4+ immune cells, pseudotyping with glycoproteins derived from other enveloped viruses is required to improve tropism for other cells. Lentiviruses pseudotyped with the attachment glycoprotein of the vesicular stomatitis virus (VSV-G) have been the most extensively investigated vectors. These vectors demonstrate significantly broadened tropism and high stability (reviewed by: [107]) and have been used to demonstrate efficient transgene delivery *in vitro* into SMCs and ECs from human saphenous vein [100], human coronary artery SMCs and ECs [108], and cardiomyocytes [109]. Comparison with Ad5 and AAV2-6 confirmed greater transgene expression in lentivirus-infected SMCs, although Ad5 was a more effective transducer of ECs [100]. Pseudotyping of lentivirus with Hantavirus glycoprotein has been shown to result in greater levels of transgene expression in the balloon-injury rabbit carotid model, and the delivery of human extracellular superoxide dismutase resulted in a reduction in neointima formation [110].

Potential clinical uses of lentivirus have been demonstrated *in vivo* in animal models. Expression of TIMP-3 resulted in reduced SMC migration and increased SMC apoptosis [100], while administration of a VSV-G pseudotyped lentivirus encoding VEGF resulted in increased angiogenesis in an *in vivo* rabbit hindlimb ischaemia model [111]. Study of direct intraventricular injection of lentivirus encoding for alpha-galactosidase in a mouse model of Fabry disease showed short-term correction of cardiac abnormalities but this benefit was lost by three months [20]. Direct intraportal injection of a third generation liver-specific lentivirus encoding for the low-density lipoprotein receptor resulted in significant reductions in serum cholesterol in a hyperlipidaemic rabbit model which were maintained up to two year follow-up [112].

Despite the potential that pseudotyped lentiviruses offer as vectors for cardiovascular gene transfer, their use in the clinical setting is very substantially hindered by concerns over their safety. The risk of insertional mutagenesis with integrative vectors has been confirmed in a clinical trial of a gammaretrovirus for the treatment of X-linked severe combined immunodeficiency. Two out of ten patients in this trial developed T-cell leukaemia as a result of integration of the vector in proximity to the LMO2 proto-oncogene [113,114]. Unlike haematological precursor cells, the cellular targets of cardiovascular gene therapy are very infrequently associated with primary neoplasia. Nonetheless there is a largely comprehensible reluctance to take risks with potentially oncogenic gene transfer vectors in any clinical setting until the potential for generation of malignancies can be shown to be within acceptable limits. The generation of replication-competent recombinant lentiviruses is also a theoretical safety concern. Non-integrating lentiviruses, created by mutation of the integrase gene, have been developed recently and offer the potential for safer gene therapy with a much lower risk of insertional oncogenesis and generation of replication-competent recombinants, whilst maintaining a broad tropism and high transduction efficiency. Despite the lack of genomic integration, long-term gene expression can occur in quiescent cells as a result of episomal nuclear retention, although the virus is inevitably lost in dividing cells. Sustained transgene expression with non-integrating lentivirus has been demonstrated *in vivo* in the rodent brain [115], retina [116], skeletal muscle [117] and liver [118]. However efficient cardiovascular gene transfer has yet to be demonstrated with integrase-deficient lentiviruses: a study with an earlier generation of integrase-defective lentivirus did not result in sustained transgene expression in cardiomyocytes [109]. For those seeking greater enlightenment, non-integrating lentiviral vectors are reviewed by Ravet *et al.* in another article in this issue.

## 4. Transcriptional Targeting

In addition to manipulating vector tropism to target those tissues to which gene delivery is desirable, it is possible to use conditional regulatory elements to confer a further level of specificity upon the manner in which gene therapies are applied to the cardiovascular system. Transcriptional targeting, by the inclusion of cell-specific promoters within the transgene expression cassette, offers the potential to increase vector safety by minimizing expression of transgene outwith specific cardiovascular cell types. Transgene expression in clinical trials has typically been driven by strong constitutively-active viral promoters. The most frequently used of these is the major intermediate-early enhancer/promoter from human cytomegalovirus (MIEhCMV), which is also the promoter most likely to be found in vectors used for studies of pre-clinical cardiovascular gene therapy [119]. Such viral promoters result in high level transgene expression in a wide variety of cell types which, although very useful for demonstrating the therapeutic potential of a transgene in short-term animal studies, is not an entirely desirable attribute for a vector to be used in clinical trials, given the potential safety concerns of ectopic transgene expression. Cell-specific promoters offer a safer means of transcriptional regulation as they preferentially drive transgene expression within a target cell and result in minimal transgene expression in other cell types. In addition, these mammalian promoters offer the potential to prolong the duration of transgene expression by reduction of the transcriptional silencing that occurs because of methylation of exogenous viral DNA sequences [120], and a lower level of immune cell transduction too [121]. Transcriptional targeting has been demonstrated to be feasible in all three of the cell types typically targeted for cardiovascular gene therapy. Unfortunately most cell-specific promoters investigated to date give rise to substantially less transgene expression in target tissues than the widely used viral promoters, and as consequence, have not seen widespread use in clinical studies of gene therapy within the vasculature.

Multiple endothelial-specific promoters have been identified including fms-like tyrosine kinase-1 (flt-1) [122], intercellular adhesion molecule-2 [123], angiopoietin-2 [124], platelet endothelial cell adhesion molecule 1 and endoglin [125]. Use of the flt-1 promoter in an adenovirus targeted to pulmonary endothelium (by use of a bi-specific Ad5 knob/angiotensin converting enzyme conjugate) conferred a very substantial improvement on specificity of transgene expression within the pulmonary vasculature than did MIEhCMV, although overall luciferase expression elicited by flt-1 was no greater than that achieved by MIEhCMV [79]. In a recent comparative study of promoters and enhancers, elements of the oxidized LDL receptor (LOX-1) promoter and the Tie2 gene enhancer in combination with an intron resulted in the highest transgene expression in rodent vascular tissue, although this expression was still less than 50% of that achieved by MIEhCMV [126].

The murine SM22α promoter regulates transgene expression in SMCs following adenovirus-mediated gene transfer *in vivo* [127], and a 999 bp sequence (−999 to −1) from the human α-SM actin promoter elicits transgene expression restricted to smooth, cardiac and skeletal muscle [128]. A short fragment of the α-SM actin promoter (−999 to −890) is responsible for enhancement of transgene expression, although in the absence of the remaining 890 bp of the sequence, the enhancer activity of this fragment is not restricted to cells of muscle lineage [128]. Unfortunately, both muscle-specific promoters elicit significantly less transgene expression in SMCs than MIEhCMV. The SM22α promoter induced ≈1,000-fold lower transgene expression in cultured vascular SMCs than MIEhCMV. The difference was less marked *in vivo*, nonetheless the SM22α promoter elicited transgene expression in ≈18-fold fewer intimal cells than MIEhCMV [129]. The 999 bp sequence from the α-SM actin promoter gave rise to ≈40% of the level of transgene expression achieved by MIEhCMV in SMCs [128]. Ribault *et al* confirmed the poor performance of the SM22α promoter, but observed that a chimeric promoter comprising a short fragment of the rabbit smooth muscle myosin heavy chain promoter [130] and the SM22α promoter improved transgene expression such that promoter activity *in vivo* approached around 25% of that of MIEhCMV and comparable biological effects were observed following use of the chimeric SMC-specific promoter or MIEhCMV to drive expression of interferon-γ in rat carotids [131]. These SMC-specific promoters face a further problem: transgene expression from both the murine SM22α and human α-SM actin promoters is significantly reduced in proliferating SMCs [132,133]. Acquisition of the proliferative phenotype typically occurs in atherosclerosis and following vascular injury, which may limit the usefulness of these promoters as regulators of transgene expression in atherosclerotic lesions and in the setting of accelerated atherosclerosis.

The ventricle-specific myosin light chain-2v promoter and the α-myosin heavy chain promoter have been demonstrated to result in cardiac specificity with both adenoviral [134–137] and AAV vectors [98,138,139] whilst the proximal human brain natriuretic peptide promoter has been shown to be effective with adenovirus [140]. Hypoxia regulatory elements (HRE) derived from the erythropoietin promoter have been employed to generate a vector from which, in combination with a constitutively active promoter, transgene expression is targeted to ischaemic tissue, including ischaemic myocardium [141]. These HRE have subsequently been combined in a chimeric construct with a fragment of the cardiac myosin light chain 2v promoter to produce a recombinant AAV that gave rise to cardiac-specific hypoxia-inducible expression of VEGF_165_ [139]. The studies of cardiomyocyte-specific promoters have been reviewed recently [26].

## 5. The State of Clinical Cardiovascular Gene Therapy

Having discussed some of the potential targets for cardiovascular gene therapy, and considered some aspects of the vectorology of cardiac and vascular gene transfer, we shall now take a brief look at the current state of cardiovascular gene therapeutics from a clinical perspective.

As we stated at the start of this article, cardiovascular diseases are the second most common target for clinical trials of gene therapy. Studies have been performed to investigate gene therapy to reduce neointima formation following PCI and for systemic cardiovascular diseases, but the large majority of clinical trials of cardiovascular gene therapy that have to date progressed to completion have investigated the induction of therapeutic angiogenesis within the peripheral vasculature and within the myocardium. Given the scope of potential targets within the cardiovascular system, it may seem initially surprising that one therapeutic objective should so dominate this field of study, but the simple fact is that the participants in these studies of angiogenesis are otherwise at a therapeutic dead-end, beyond further percutaneous or operative intervention, with only limb amputations or persistent symptomatic myocardial ischaemia (with the incumbent impositions on quality of life that these burdens convey) to countenance.

Despite the preponderance of studies of viral gene transfer in the pre-clinical setting, a substantial proportion of clinical trials of cardiovascular gene therapy have employed non-viral gene transfer in preference to virus-mediated methods. As the remit of this article is confined to virus-mediated gene therapy, we shall defer from comment on such clinical studies of plasmid-mediated gene delivery. The reader who is interested in the outcomes of the those clinical trials in which non-viral means of gene transfer were employed is referred to the review by Rissanen and Yla-Herttuala, which nicely summarizes the state of play in early 2007 [142].

### 5.1. Angiogenic Gene Therapy

The induction of angiogenesis as a therapeutic strategy for both coronary and peripheral arterial disease has been investigated in a series of clinical randomised clinical trials, primarily using vascular endothelial growth factor (VEGF) or fibroblast growth factor (FGF) as the proangiogenic transgene. Most research on PAD has employed plasmid vectors for gene transfer, although studies of viral gene therapy have been published: in a phase II study of intra-arterial injection of AdVEGF_165_ following peripheral angioplasty an increase in new vessels distal to the site of vector delivery was demonstrated, but this was not accompanied by improved healing of ischaemic ulcers, resolution of rest pain or increased ankle-brachial index by comparison with controls [143]. Interestingly, in the same study, a similar effect was elicited by plasmid/liposome delivery of VEGF_165_ as was achieved by AdVEGF_165_. In the RAVE study of intramuscular injection of AdVEGF_121_, there was no improvement in measures of ischaemia or clinical outcomes, although the therapy was well-tolerated [144]. The lack of demonstrable, clinically beneficial effect from adenovirus-mediated gene transfer in the periphery appears to have largely silenced further interest in virus-mediated gene transfer in this setting, although an ongoing phase II study of Ad2-mediated delivery of hypoxia inducible factor-1α (entitled WALK) is expected to deliver results in 2010 (http://clinicaltrials.gov/ct2/show/NCT00117650), and it will be of enormous interest to see if this study offers something of greater therapeutic substance than has been reported previously in this setting.

In contrast to studies of PAD, the majority of studies of angiogenic gene transfer for myocardial ischaemia have used virus-mediated (specifically, adenovirus-mediated) gene transfer – presumably it is easier to convince regulatory authorities of the life-saving potential of viruses in this setting than in the limb vasculature (patients with PAD do not typically die of their peripheral arterial problems, but of myocardial infarction or stroke).

In the earliest study of intracoronary injection of AdFGF-4 (AGENT-2), delivery of active vector by subselective coronary catheterization of culprit arteries resulted in a borderline significant reduction in the size of the region of myocardium demonstrating stress hypoperfusion eight weeks post-delivery [145]. This was not accompanied however, by a significant clinical effect. At around the same time, the KAT study investigated localized intracoronary delivery of AdVEGF_165_. This study differed from AGENT-2 in employing an intracoronary balloon catheter to attempt to restrict vector delivery to the coronary artery wall at the site of stent deployment, rather than simply injecting adenoviruses down the coronary artery. As was the case in the AGENT-2 study however, no clinical effect was observed, and no effect was evident on in-stent restenosis either although an improvement was claimed in myocardial perfusion as assessed by cardiac SPECT imaging at six months post-delivery when compared with the pre-PCI myocardial perfusion in the AdVEGF_165_-treated group [146]. The data presented however do not actually suggest that there was a significant difference in myocardial perfusion between the treated and control groups at 6 months after PCI and vector delivery.

Further studies of intracoronary injection of AdFGF-4, in the guise of the AGENT-3 and AGENT-4 studies, have given rise to the largest experience to date of cardiovascular gene therapy, with over 500 patients with chronic angina having undergone enrolment [147]. Intracoronary administration of Ad5-FGF-4 failed to improve the primary end-point of total exercise time. However further analysis of these trials has identified unusual gender-specific results, with a significant improvement in clinical outcomes (exercise treadmill endurance and angina class) for women in a vector-dose-related fashion at both six and twelve months post-delivery, with greater improvements observed in those exposed to a greater vector dose. No significant improvement was observed in men. This finding is being investigated further in the phase III AWARE trial (http://clinicaltrials.gov/ct2/show/NCT00438867).

In addition to intracoronary administration of viruses, direct intramyocardial injection of adenovirus has been investigated following thoracotomy in human studies. Administration of Ad5-VEGF_121_ hinted at clinical efficacy in a phase I study, with “suggested” improvements in regional ventricular wall motion, severity of angina and exercise tolerance [148]. This led to the phase II REVASC study which reported improvements in the primary end-point of exercise time as compared to a medical therapy control group who did not undergo thoracotomy. However myocardial perfusion was significantly worse in the treatment group and it is likely that the improvement in symptoms in this trial was largely attributable to a placebo effect related to the thoracotomy [149].

All considered, adenovirus-mediated myocardial gene transfer of pro-angiogenic genes has provided little cause for enthusiasm about the potential for widespread clinical application of such therapy. However, the fact that it has been possible to elicit clinically useful effects (albeit restricted to women) by delivery of adenoviruses, which – it must be remembered – are likely to give rise to appreciable transgene expression for only two or three weeks post-delivery, does raise the possibility that more useful clinical effects may arise from the use of vectors that will elicit transgene expression of longer duration. In this respect, it will be interesting to see whether studies of angiogenic gene transfer using AAV, gutless adenoviruses or lentivirus will ever see the light of day.

### 5.2. Reduction of Neointima Formation

A vast number of potentially therapeutic transgenes have been studied in animal models of restenosis [91] but very few clinical trials have been performed. The KAT trial, which was discussed in the previous section, assessed restenosis as a secondary endpoint following local virus delivery to the site of coronary angioplasty (92/103 of patients received stents) [146]. Administration of AdVEGF_165_ had no effect on angiographic restenosis at six months. Similarly, in the study by Laitinen and colleagues, AdVEGF_165_ improved angiogenesis but did not reduce restenosis at the site of peripheral angioplasty [143].

Gene-eluting stents using both adenovirus and plasmid vectors have been investigated *in vivo* [6,7,150,151]. Reductions in neointima formation have been demonstrated following adenovirus-coated stent implantation in the rat carotid [6,7] and rabbit iliac arteries [150] but no human trials of this method of vector delivery have been performed at the time of writing. In truth, the relative success of drug-eluting stents (DES) at preventing (or at least delaying the onset of) in-stent restenosis really means that the technical demands of virus-mediated gene therapy are never going to be suited to stent-mediated delivery in the clinical setting. DES can be deployed into a patient straight out of the packet without any time-consuming virus loading (and concomitant biological safety paraphernalia) and any competing technology must offer a similar ease of use for the clinician or some very substantial clinical benefit. In this respect, virus-mediated stent-based gene transfer is akin to intra-coronary brachytherapy in that potential benefits exist (albeit in the case of virus-mediated gene transfer, never proved in the clinical setting), but those benefits are offset by the technical difficulties incumbent upon actually delivering the therapy. Any gene therapeutic approach to ISR must be as easy to deliver clinically as a DES, which means plasmid-eluting stents may be a preferable option.

*Ex vivo* delivery of virus to vein grafts at the time of CABG offers a more immediately tempting milieu for virus-mediated vascular gene therapy than ISR [152] and efficacy in pre-clinical models has been demonstrated using many transgenes [153,154]. However, in spite of this, no clinical studies using *ex vivo* viral gene delivery to human saphenous vein bypass conduits have been reported yet. The only clinical trial of nucleic acid therapy aimed at amelioration of bypass graft disease (using an elongation factor 2 transcription decoy oligonucleotide) reported negative results [155]. Part of the difficulty underlying translation of pre-clinical work into the real world of cardio-thoracic surgery is the short-term nature of those pre-clinical studies that have been performed: vein-graft disease is a phenomenon that manifests clinically over years rather than weeks or months, and it is almost certain that to be effective gene therapy strategies will have to elicit transgene expression for years too. Low-generation adenoviruses, the vector used in the large majority of pre-clinical studies of vein graft NIH, are entirely unsuitable for long-term gene transfer, and those vectors that *are* suited for this purpose (gutless adenovirus, AAV, lentivirus [100,156]) have scarcely been studied in this setting; only one study of “therapeutic” gene transfer employing any of these vectors in a model of vein graft disease has made it to press as far the authors are aware [157]. Furthermore, means of delivering plasmid DNA with sufficient efficacy to be of potential clinical value after clinically-pertinent periods of exposure are surfacing [158], so it is distinctly possible that non-viral gene transfer will eventually displace virus-mediated gene transfer as the most clinically-relevant method of gene transfer in this setting too.

### 5.3. Gene Therapy for Heart Failure

The only completed clinical trials of myocardial gene therapy to date have been of proangiogenic factors, and as already discussed, the outcomes of these studies have been less than impressive. However myocardial gene therapy has recently become the focus of renewed interest due to the initiation of clinical studies using an AAV vector for the treatment of heart failure. Two excellent recent reviews discuss this field in more detail [24,159]. Briefly, the SERCA2a gene encodes for the sarcoplasmic reticulum calcium ATPase pump which transfers cytoplasmic calcium back into the sarcoplasmic reticulum during cardiomyocyte relaxation. A decrease in activity of SERCA2a and subsequent impaired calcium reuptake has been shown to be present in human heart failure [160] and animal models have demonstrated that transgene expression of SERCA2a using viral vectors (adenovirus and AAV) can improve left ventricular function [161]. As a result of promising preclinical data, two studies of gene therapy in heart failure have received approval using AAV vectors containing the human CMV promoter and the SERCA2a transgene. The CUPID study is a phase 1/2 placebo-controlled clinical trial and randomised patients with severe heart failure of either ischaemic or non-ischaemic aetiology to receive either a ten minute intracoronary infusion of AAV1-CMV-SERCA2a or placebo. The study has finished recruitment and preliminary results are expected in 2010 [162]. A second study in the UK is investigating gene delivery of the same transgene using a different AAV serotype (AAV6-CMV-SERCA2a) in patients with end-stage heart failure who have already undergone left ventricular assist device (LVAD) implantation. This trial has received approval and is due to commence recruitment in early 2010 (http://clinicaltrials.gov/ct2/show/NCT00534703).

It is expected that these studies will provide important information on both the suitability of AAV as a vector and whether SERCA2a is a beneficial transgene in human heart failure. Although the results are eagerly awaited there are several reasons to be cautious. The vectors chosen for these studies may not be the optimal vectors for myocardial gene delivery; as discussed earlier, the AAV8 and AAV9 serotypes have been shown to exhibit greater myocardial tropism than the AAV1 and AAV6 serotypes, and the human CMV promoter, whilst efficacious in cardiomyocytes, runs the risk of being rendered quiescent in the long-term as a consequence of DNA methylation. There are also some safety concerns with the use of SERCA2a. Overexpression of SERCA2a in rat myocardium leads to an increased rate of fatal arrhythmia [163] and for this reason all patients in the CUPID trial are required to have an implantable cardiac defibrillator prior to enrolment. Still, this is a therapeutic area where there is a great (and growing) clinical need and it represents one of the cardiovascular targets where gene therapy can hope to offer something entirely new, although it is important not to pin too many hopes on a successful outcome in what is effectively the first therapeutic iteration in this area.

## 6. Conclusions

It is difficult to write a review of gene therapy with regard to any of its spheres of application without being constrained eventually to resort to talk of its “promise” or “potential” in the clinical translation of that setting; a brief scout through PubMed reveals that the oldest hit for “gene therapy” and “promise” dates from as far back as 1981 [164]. In the intervening decades there has been remarkable progress in vector development, and pre-clinical (e.g., animal) studies have revealed literally hundreds of *potentially* therapeutic transgenes that might be applied to the cardiovascular system, but we have yet, 28 years after the first uttering of the promise of gene therapy, to see any genuine clinically-useful outcome from the cardiovascular application of those transgenes that have offered us *promise* in the pre-clinical setting. So why is that and what can we do about it?

Firstly, it is possible that we have been misled about the potential for gene therapy. The talk of promise and potential is, after all, based upon pre-clinical studies. Until some unequivocal clinical benefit is obtained from the extrapolation of results obtained *in vivo*, there will always be some doubt about the validity of attempting to translate observations made in pre-clinical models into the real world. In many respects this cannot be avoided and even the very best pre-clinical models are poor surrogates for the disease processes that affect man. Perhaps then, we have yet to study a virus-mediated gene therapy in the vasculature that will actually afford any benefit in man. It seems unlikely, but there is little evidence as yet to suggest the contrary. This should not stop us looking, of course.

At the present, efficient gene transfer in clinically-applicable exposure times is typically reliant upon the use of viruses (although gene therapy for peripheral arterial disease is an exception in this regard and much of the currently ongoing clinical research uses non-viral gene transfer), and those viruses that are available (or at least those that have been used in clinical trials of cardiovascular diseases) are either potently immunogenic and pro-inflammatory or difficult to generate at high titres. This naturally begs the question, why have investigators not used better vectors for clinical gene transfer? Why have there, alluding specifically to the cardiovascular system, been no studies of clinical gene transfer using helper-dependent adenoviruses, for example? Gutless adenoviruses offer a lot of very tempting features as clinical gene transfer vectors. They can be expected to elicit transgene expression of similar magnitude to that achieved by first-generation vectors, but for longer and with less concomitant inflammation, yet so far the authors are aware of no plans to use them in a clinical setting. Similarly, the two recent studies of AAV-mediated gene transfer of SERCA2a have employed (or will employ) AAV serotypes that, whilst effective for use in cardiomyocyte gene transfer, are not the most effective vectors for this purpose. Once again, it isn’t immediately apparent why clinical investigators are relying on suboptimal gene transfer strategies. It might be argued, of course, that we simply don’t know if the methods being employed *are* suboptimal in the clinical setting, but if we are to derive maximum value from the results of pre-clinical studies it surely resides in allowing us to anticipate best practice when those strategies that have been investigated in animals are translated into man.

It may be that this problem is more closely related to commercial issues than technical matters. Compared to classical pharmacological agents (with regard to which a lone company can generate, develop, investigate and eventually market a unique molecule) gene therapy agents are complex. Multiple components are required to generate a vector that can be delivered to a target tissue, and give rise to expression of a therapeutic protein in humans. The simplest virus vector requires the therapeutic transgene itself, a promoter of transgene expression, a polyadenylation signal and the apparatus necessary to package them into the chosen viral agent. This typically represents four separate patented technologies and four separate parties who have to be reconciled with the development of a product for clinical trials. Against this backdrop it should perhaps come as no surprise that not all technologies are available for all investigators, and it is probably not adopting too cynical a posture to say that the widely dispersed intellectual properties that underlie viral gene therapy vectors are a discouragement to the involvement of big pharmaceutical companies in clinical trials of these agents. Until it becomes clear that gene therapies can be transferred effectively into the clinic with profit-making potential, clinical trials of gene therapy are likely to be forced to accept compromises in their implementation.

Another of the problems that gene therapies face in the cardiovascular setting is the need to compete in many therapeutic areas with classical pharmacological therapies that are, in fact, adequate (albeit sometimes little more than adequate) for their clinical purposes. Gene therapy, and particularly virus-mediated gene therapy, has still to contend with the widely-held belief that it is a risky therapeutic approach to a problem comparable, for example, with systemic chemotherapy. This is reinforced by infamous failures of the past: the most widely-known recipient of virus-mediated gene therapy was the first person to die as a direct consequence of the agent he received. Speaking from personal experience, many experienced medical practitioners who know almost nothing else of gene therapy are aware that Jesse Gelsinger died as a result of a large dose of recombinant adenovirus vector. Perhaps because of this gene therapies tend still to be reserved for application to clinical situations in which no adequate classical pharmacological therapies exist. Certainly within the cardiovascular system, trials of gene therapy have been predominantly directed at angiogenesis in patients who are symptomatic despite best medical care and for whom revascularization is no longer a viable option. There can be no doubt that a therapy that proves successful in these patients will be very welcome indeed, but the fact that these patients have exhausted those therapies that are currently available does mean that a lot is being asked of gene therapy to succeed where well-established therapies have failed. In that respect, it seems that cardiovascular gene therapies are going to have to be proved successful in some of the most very demanding of clinical situations before they are applied to settings where they might more reasonably be expected to achieve clinical usefulness.

The perception of gene therapy as a high-risk option naturally accounts for the preponderance of clinical studies that are aimed at treating malignant diseases. It ought to be stressed however that the prognosis of certain cardiovascular diseases, severe left ventricular dysfunction for example, is not so very different from that of some malignancies and, while drug treatments are available that modify the disease course and prolong life expectancy of patients with heart failure, there are no cures for the large majority of patients. As such these patients are in need of novel therapies to no lesser extent than are victims of cancer. Where one of the most important differences between severe heart failure and cancer, however, is that once the cellular targets of oncological gene therapies have been successfully targeted there is no virtue in persistence of gene expression, as the purpose of gene expression is to kill the cell in which expression occurs. In contrast, some cardiovascular targets for gene therapy may require long-term transgene expression. Studies of cancer gene therapy can therefore make effective use of simple, easy-to-produce vectors such as first-generation recombinant adenoviruses, which are not likely to find a useful clinical application in cardiovascular gene therapy. Certainly, the evidence available to date shows that very little clinical benefit has accrued in any cardiovascular setting from use of first-generation adenoviruses.

As a result of the issues discussed above, successful virus-mediated gene therapy for cardiovascular disease is harder to achieve than, say, cancer gene therapy, and the rewards are often regarded as being less. Despite this, at the time of writing, recombinant viral vectors still offer the greatest potential to apply gene therapy strategies to cardiovascular disease and it is new developments in viral vectors that are likely to reap the earliest rewards in the clinical setting. Vectors that are available now (for instance pseudotyped AAV), despite the reservations voiced above, offer the realistic proposition of myocardial gene therapies for heart failure. The first iteration of these therapies is not going to be the definitive iteration thereof (as is always the case when new technologies are applied to therapeutics) and the population being studied in these early trials manifest extreme degrees of the disease state, so it is unrealistic to expect dramatic benefits, but any beneficial effect will be a step forward.

Similarly, despite the problems inherent in the application of recombinant adenoviruses to the cardiovascular system, the *ex vivo* opportunities afforded by vein graft gene transfer represent a target in which adenoviruses, in the guise of pseudotyped or targeted gutless vectors, are likely to offer some therapeutic value. The manufacture of these vectors is well within the remit of currently available technologies. There are some applications which are likely never to be suitable for virus-mediated gene transfer: virus delivery to stented coronary arteries is not going to displace DES for instance, but there are also pathologies for which pre-clinical efficacy has been demonstrated (for example, hypercholesterolaemia [112], biopacemaking [19], hypertension [165]) and which are simply waiting for the right vector to become available to study in the clinical setting.

Whilst at the present all that gene therapy has to offer in the setting of cardiovascular disease is potential or promise (whichever is your preference), the prospect is implausible that gene therapies will not at some time in the future become a routine part of everyday therapeutics. And while it is equally likely that when this time arrives we will not be using virus vectors as we recognize them today, recombinant viruses represent the most important pathway by which gene therapy will gain initial credibility as a therapeutic modality in the setting of cardiovascular disease. Viruses have taken millions of years to evolve the means by which they deliver their nucleic acids to the nuclei of the cells that they infect. Gene therapists might reasonably ask for something more than 28 years to turn the promise that virus–mediated gene transfer offers into effective gene therapies.

## Figures and Tables

**Table 1.** Choice of vector for clinical application.

**Clinical application**	**Potential delivery method**	**Desired onset of gene expression**	**Desired duration of gene expression**	**Target cells**	**Possible vectors**	**Desirability for repeat dosing**
In-stent restenosis	Intracoronary infusion/bound to stent	Rapid	Weeks-Months	Vessel wall (SMC, EC)	Plasmids Adenovirus	+++
SVG degeneration	*Ex vivo* direct application	Rapid	Weeks-Months	Vessel wall (SMC, EC)	Adenovirus AAV Lentivirus Plasmids	++
Heart failure	Intracoronary infusion; myocardial injection	Not important	Months-permanent	Cardiomyocytes	AAV Lentivirus	+
Cardiac angiogenesis	Intracoronary infusion; myocardial injection	Not important	Weeks-months	Vessel wall (EC)	AAV Lentivirus Adenovirus	+
Peripheral angiogenesis	Intra-arterial infusion; intramuscular injection	Not important	Weeks-months	Vessel wall (EC)	Plasmids Adenovirus AAV Lentivirus	+
Hypercholesterolaemia	Intravenous	Not important	Permanent	Hepatocytes	AAV Lentivirus Adenovirus	-
Biopacemaking	Myocardial injection; coated on pacing wires	Rapid	Permanent	Cardiomyocytes	Lentivirus AAV Plasmids	-

## Abbreviations

AAV: Adeno-associated virus
Ad: Adenovirus
CABG: Coronary artery bypass grafting
CAD: Coronary artery disease
CVS: Cardiovascular system
DES: Drug eluting stents
EC: Endothelial cell
ECM: Extracellular matrix
ISR: Instent restenosis
NIH: Neointimal hyperplasia
PAD: Peripheral arterial disease
PCI: Percutaneous coronary intervention
SMC: Smooth muscle cell
SVG: Saphenous vein graft
